# *UniEuk*: Time to Speak a Common Language in Protistology!

**DOI:** 10.1111/jeu.12414

**Published:** 2017-04-21

**Authors:** Cédric Berney, Andreea Ciuprina, Sara Bender, Juliet Brodie, Virginia Edgcomb, Eunsoo Kim, Jeena Rajan, Laura Wegener Parfrey, Sina Adl, Stéphane Audic, David Bass, David A. Caron, Guy Cochrane, Lucas Czech, Micah Dunthorn, Stefan Geisen, Frank Oliver Glöckner, Frédéric Mahé, Christian Quast, Jonathan Z. Kaye, Alastair G. B. Simpson, Alexandros Stamatakis, Javier del Campo, Pelin Yilmaz, Colomban de Vargas

**Affiliations:** ^1^ Sorbonne Universités UPMC Université Paris 06 & CNRS, UMR7144Station Biologique de RoscoffPlace Georges TeissierRoscoff29680France; ^2^ Department of Life Sciences and ChemistryJacobs University gGmbHBremenD‐28759Germany; ^3^ Gordon and Betty Moore Foundation1661 Page Mill RoadPalo AltoCalifornia94304USA; ^4^ Department of Life SciencesNatural History MuseumCromwell RoadLondonSW7 5BDUnited Kingdom; ^5^ Geology and Geophysics DepartmentWoods Hole Oceanographic InstitutionWoods HoleMassachusetts02543USA; ^6^ Division of Invertebrate Zoology & Sackler Institute for Comparative GenomicsAmerican Museum of Natural HistoryNew YorkNew York10024USA; ^7^ European Nucleotide Archive, EMBL‐EBIWellcome Genome CampusCambridgeCB10 1SDUnited Kingdom; ^8^ Department of Botany and ZoologyUniversity of British Columbia109‐2212 Main MallVancouverBCV6T 1Z4Canada; ^9^ Department of Soil Sciences, College of Agriculture and BioresourcesUniversity of SaskatchewanSaskatoonSKS7N 5C5Canada; ^10^ Centre for Environment, Fisheries and Aquaculture ScienceBarrack RoadWeymouthDT4 8UBUnited Kingdom; ^11^ Department of Biological SciencesUniversity of Southern California3616 Trousdale ParkwayLos AngelesCalifornia90089‐0371USA; ^12^ Scientific Computing GroupHeidelberg Institute for Theoretical StudiesSchloss‐Wolfsbrunnenweg 35HeidelbergD‐69118Germany; ^13^ Department of EcologyUniversity of KaiserslauternKaiserslauternD‐67663Germany; ^14^ Department of Terrestrial Ecology, Netherlands Institute of Ecology (NIOO‐KNAW) & Laboratory of NematologyWageningen UniversityDroevendaalsesteeg 10Wageningen6708 PBThe Netherlands; ^15^ Microbial Genomics and Bioinformatics Research GroupMax Planck Institute for Marine MicrobiologyCelsiusstrasse 1BremenD‐28359Germany; ^16^ CIRAD, UMR LSTMMontpellierF‐34398France; ^17^ Department of BiologyDalhousie University1355 Oxford StreetHalifaxNSB3H 4R2Canada; ^18^ Karlsruhe Institute of Technology, Institute for Theoretical InformaticsPostfach 6980Karlsruhe76128Germany

**Keywords:** Community expertise, diversity, eukaryotes, *EukBank*, *EukMap*, *EukRef*, taxonomy

## Abstract

Universal taxonomic frameworks have been critical tools to structure the fields of botany, zoology, mycology, and bacteriology as well as their large research communities. Animals, plants, and fungi have relatively solid, stable morpho‐taxonomies built over the last three centuries, while bacteria have been classified for the last three decades under a coherent molecular taxonomic framework. By contrast, no such common language exists for microbial eukaryotes, even though environmental ‘‐omics’ surveys suggest that protists make up most of the organismal and genetic complexity of our planet's ecosystems! With the current deluge of eukaryotic meta‐omics data, we urgently need to build up a universal eukaryotic taxonomy bridging the protist ‐*omics* age to the fragile, centuries‐old body of classical knowledge that has effectively linked protist taxa to morphological, physiological, and ecological information. *UniEuk* is an open, inclusive, community‐based and expert‐driven international initiative to build a flexible, adaptive universal taxonomic framework for eukaryotes. It unites three complementary modules, *EukRef*,* EukBank*, and *EukMap*, which use phylogenetic markers, environmental metabarcoding surveys, and expert knowledge to inform the taxonomic framework. The *UniEuk* taxonomy is directly implemented in the European Nucleotide Archive at EMBL‐EBI, ensuring its broad use and long‐term preservation as a reference taxonomy for eukaryotes.

THE bewildering organismal and functional complexity of microbial eukaryotes has long fascinated protistologists but exceeded the capacity of this research community to comprehensively study it. Lacking the critical mass for a strong scientific discipline, protistologists remain largely divided into various sub‐communities (protozoology versus phycology, aquatic versus terrestrial systems, fossil versus extant organisms, etc.), many of which are adjuncts of other larger fields that may speak different technical languages or use different taxonomic systems. Today, environmental ‘‐omics’ surveys make it possible to explore the boundaries of the total biotic diversity in ecosystems, from viruses to animals (Bork et al. 2015). These studies indicate that microbial eukaryotes comprise a huge amount of the organismal and genetic complexity of our planet's biomes, potentially even the majority (e.g. Mahé et al. 2017; de Vargas et al. 2015). This discovery increases the challenge of studying the full complexity of protists, but, at the same time, provides an exceptional opportunity to unite and strengthen the field of modern protistology. For this to occur, it is paramount to construct a universal taxonomy for eukaryotes, a common language that will help unify the field and connect the deluge of new molecular‐genetic datasets with each other and with the centuries of accumulated morphological, physiological, life history, and ecological information on these organisms.

*UniEuk* (http://www.unieuk.org/) is an open, inclusive, community‐based and expert‐driven international initiative to build a flexible, adaptive universal taxonomic framework for eukaryotes that represents the views of the research community. The effort is focused primarily on protists, and can also incorporate existing taxonomic systems for animals, plants, and fungi. Organism‐based and informed by phylogeny, the *UniEuk* framework integrates expert knowledge about morphology and ecology with key molecular information from phylogenetics and environmental ‘‐omics’ surveys to capture our total current knowledge on eukaryotic diversity, evolution, and ecology. Its power resides primarily on bottom‐up community efforts organized around all protist clades to capture collective knowledge on eukaryotic diversity, with validation of the taxonomic framework by an extensive network of experts. The system's broad use and preservation will be ensured by a direct implementation of the *UniEuk* taxonomy into the European Nucleotide Archive (ENA) at EMBL‐EBI (http://www.ebi.ac.uk/ena), with the long‐term goal of becoming the reference taxonomy in all INSDC genetic data repositories.

*UniEuk* was launched in May 2016 with initial funding from the Gordon and Betty Moore Foundation (http://www.moore.org) and the International Society of Protistologists. During the first year, the project's taxonomy and database coordinators and members of the *UniEuk* Steering, Advisory, and Technical Committees (http://www.unieuk.org/people/) designed the three main complementary and interconnected modules for direct community interaction—*EukRef*,* EukBank*, and *EukMap*—that together constitute the core of the *UniEuk* system and are necessary to build the universal taxonomic framework (below and Fig. 1). They also established a baseline version of the *UniEuk* taxonomic framework, starting from existing systems (e.g. Adl et al. 2012), and integrating the most up‐to‐date information from phylogenomic evidence (e.g. Burki et al. 2016). Lastly, they proposed a set of guidelines for naming environmental genetic lineages prior to their morphological characterization.

**Figure 1:**
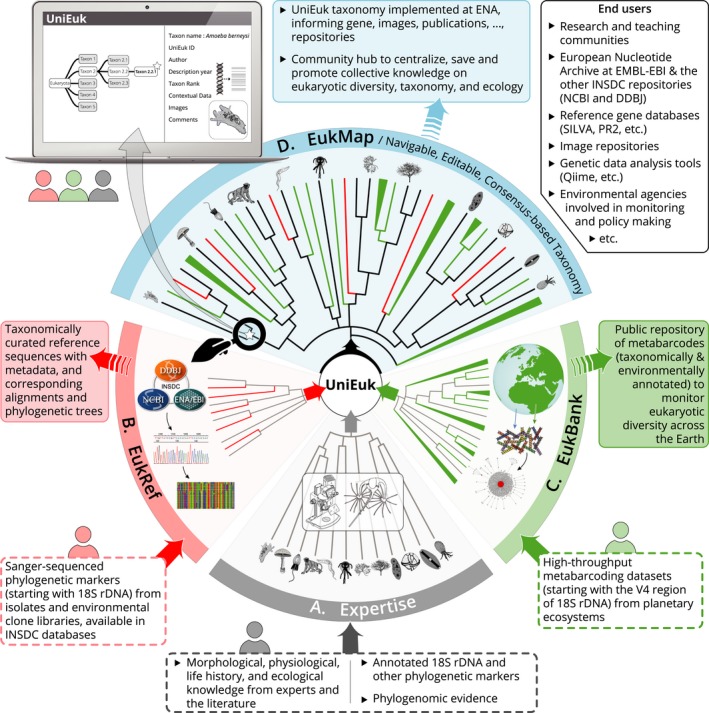
The *UniEuk* workflow. Bottom‐up, community‐based information on eukaryotic biodiversity from (A) classical knowledge, (B) phylogenetic diversity, and (C) environmental ‘‐omics’ surveys, converge and synergize through the *UniEuk* modules to inform the navigable and editable, consensus‐based taxonomic framework (D). Dotted and colored frames indicate input and output information, respectively. Line drawings of eukaryotes adapted with permission from https://genev.unige.ch/system/pawlowski/lab/tree.png.

1. *EukRef* (Fig. 1B): The *EukRef* module allows integration into the *UniEuk* system of all preexisting phylogenetic information on eukaryotic diversity derived from Sanger‐sequenced DNA markers of described taxa and environmental clones (beginning with 18S rDNA sequences longer than 500 bp). *EukRef* uses a standardized, open‐source bioinformatics pipeline to generate homogenous, high‐quality curation of 18S rDNA sequences available in the INSDC databases. *EukRef* outputs include, on a lineage‐specific basis, taxonomically curated 18S rDNA sequences with corresponding sequence alignments and phylogenetic trees. In addition to being a direct source of information for the *UniEuk* taxonomic framework, *EukRef* outputs represent stand‐alone community resources shared through partner 18S rDNA reference databases SILVA (Quast et al. 2013) and PR^2^ (Guillou et al. 2013). *EukRef* has thus far largely engaged PhD students and postdocs who learned how to use the pipeline during multiday workshops, progressively expanding to include all eukaryotic clades. Information on *EukRef* and on past and future workshops can be found at http://www.eukref.org/.
2. *EukBank* (Fig. 1C): The *EukBank* module allows integration into the *UniEuk* system of the enormous and largely nameless genetic information on eukaryotic diversity obtained from high‐throughput metabarcoding (HTM) surveys of the Earth's ecosystems. Combining an ultra‐fast algorithm generating stable clusters of amplicons (Mahé et al. 2015) and state‐of‐the‐art methods of phylogenetic placement (Berger et al. 2011), *EukBank* will absorb and reduce the complexity of eukaryotic HTM datasets, and analyze them phylogenetically. Datasets from all planetary biomes will be incorporated, starting with the V4 18S rDNA marker (Pawlowski et al. 2012). *EukBank* is centralized at ENA, providing the community with a protocol and direct assistance for the submission of HTM datasets and their critical metadata to the repository. *EukBank* will allow monitoring of total eukaryotic diversity (e.g. saturation, phylogeny) across biomes, as well as identification and preliminary naming of novel eukaryotic lineages of ecological and/or phylogenetic relevance. These will be integrated into the *UniEuk* taxonomic framework, thus highlighting parts of the tree of eukaryotic life warranting deeper investigation. *EukBank* is aimed at all scientists who have generated eukaryotic HTM datasets and are interested in discovering how these contribute to a growing global perspective on eukaryotic diversity.
3. *EukMap* (Fig. 1D): The *EukMap* module allows the community to directly interact with and inform the growing universal taxonomic framework. *EukMap* is a user‐friendly representation of the *UniEuk* taxonomic framework, publicly navigable, where each node/taxon is associated with standardized features (name, contextual data, links to representative pictures, etc.). *EukMap* will integrate curated genetic information from *EukRef* and *EukBank*, and represents a community hub to centralize, safeguard, and promote our current global knowledge on eukaryotic diversity, taxonomy, and ecology. The output of *EukMap* (the actual *UniEuk* taxonomy) will be directly applied to ENA at EMBL‐EBI, with regular versioning. It will provide continuous feedback to the other *UniEuk* modules and partner reference gene databases for optimized and standardized taxonomic annotation of environmental sequence data. It will also be useable as a stand‐alone summary of the collective knowledge on eukaryotic diversity and evolutionary history for scientific, educational, or public outreach purposes. *EukMap* is aimed at all researchers, from students to professors, with expertise on eukaryotic taxonomy, ecology, and evolution. The taxonomic framework as a whole is freely navigable for all visitors. Once registered into the *UniEuk* system, community members will be able to propose changes and addition of missing taxa or contextual information (including images) at any taxonomic level, and participate in group‐specific discussions to reach agreement on which changes should be adopted in official releases of the *UniEuk* taxonomy. Lead taxonomy experts and the project taxonomy and database coordinators will be in charge of moderating these discussions and implementing decisions.

Further information on *UniEuk*'s vision, goals, and organization can be found at http://www.unieuk.org/, together with use‐case scenarios and FAQs, a news section, and a registration page. We encourage all scientists with expertise in protist taxonomy, ecology, or evolution to join the growing *UniEuk* community (Box 1). The deluge of emerging eukaryotic genetic data makes this the right time to build a common, eco‐morpho‐genetic and organism‐centered language for protistology. With its complementary, yet independent modules (all with stand‐alone outputs benefitting end‐users in the protistology community and beyond; see Fig. 1), we believe that *UniEuk* will generate the necessary momentum to address this challenge. Crucially, the direct application of the *UniEuk* taxonomic framework to all INSDC sequence records via the ENA node at EMBL‐EBI means that your contributions will have a direct, immediate impact on global eukaryotic research efforts worldwide. Together, let us make the 21st century the age of protistology!

Box 1 **UniEuk timeline and ICOP 2017 announcement**

The three main *UniEuk* modules are in the process of implementation. The full *UniEuk* system will be demonstrated to the community at the next joint International Congress of Protistology (ICOP 2017) and Annual Meeting of the International Society of Protistologists in Prague, Czech Republic, July 30 to August 4, 2017 (http://www.icop2017.org/). We encourage all congress participants to attend the *UniEuk* session that will take place the afternoon of Thursday August 3, 2017. After this, the project will be ready to interact with those of you not yet involved: especially experts in protist biodiversity and taxonomy! Visit our website to preregister and be kept updated when the various functionalities of the system become available (http://www.unieuk.org/register/).
